# Correction: Iridium-catalyzed enantioselective olefinic C(sp^2^)–H allylic alkylation

**DOI:** 10.1039/d1sc90022f

**Published:** 2021-02-11

**Authors:** Rahul Sarkar, Santanu Mukherjee

**Affiliations:** Department of Organic Chemistry, Indian Institute of Science Bangalore 560 012 India sm@iisc.ac.in +91-80-2360-0529 +91-80-2293-2850

## Abstract

Correction for ‘Iridium-catalyzed enantioselective olefinic C(sp^2^)–H allylic alkylation’ by Rahul Sarkar *et al.*, *Chem. Sci.*, 2021, DOI: 10.1039/d0sc06208a.

The authors regret a minor error in Scheme 3(B), the corrected figure is shown below.

**Scheme 3:**
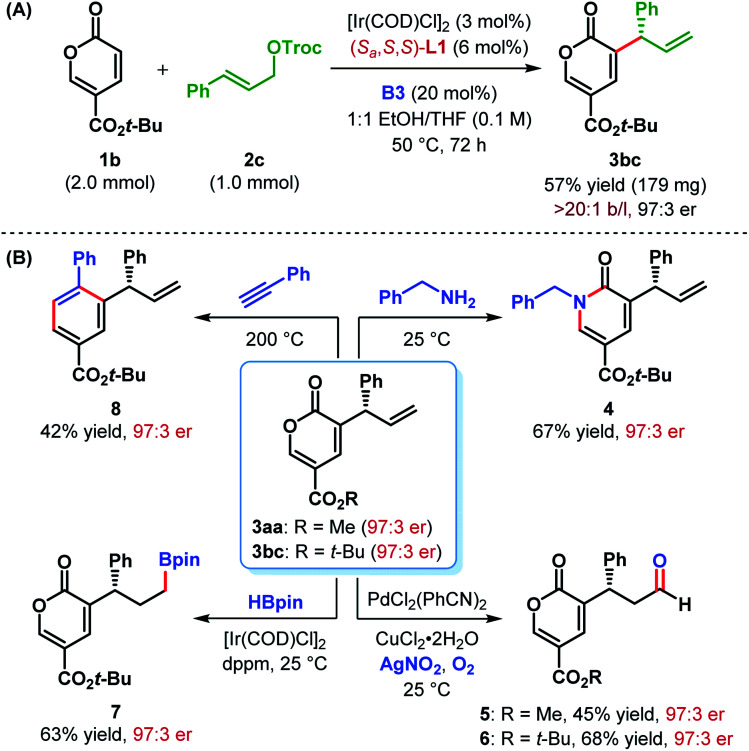
(A) Scale-up of α-C(sp^2^)–H allylic alkylation and (B) synthetic elaborations of α-allyl-coumalates.

The Royal Society of Chemistry apologises for these errors and any consequent inconvenience to authors and readers.

